# Metformin for early comorbid glucose dysregulation and schizophrenia spectrum disorders: a pilot double-blind randomized clinical trial

**DOI:** 10.1038/s41398-021-01338-2

**Published:** 2021-04-14

**Authors:** Sri Mahavir Agarwal, Roshni Panda, Kenya A. Costa-Dookhan, Nicole E. MacKenzie, Quinn Casuccio Treen, Fernando Caravaggio, Eyesha Hashim, General Leung, Anish Kirpalani, Kelly Matheson, Araba F. Chintoh, Caroline K. Kramer, Aristotle N. Voineskos, Ariel Graff-Guerrero, Gary J. Remington, Margaret K. Hahn

**Affiliations:** 1 Centre for Addiction and Mental Health, Toronto, ON Canada; 2 Department of Psychiatry, University of Toronto, Toronto, ON Canada; 3 Institute of Medical Science, Faculty of Medicine, University of Toronto, Toronto, ON Canada; 4 St. Michael’s Hospital, Toronto, ON Canada; 5 Mount Sinai Hospital, Toronto, ON Canada; 6 Department of Medicine, Division of Endocrinology and Metabolism, University of Toronto, Toronto, ON Canada; 7 Banting and Best Diabetes Centre, University of Toronto, Toronto, ON Canada

**Keywords:** Physiology, Schizophrenia

## Abstract

Patients with schizophrenia have exceedingly high rates of metabolic comorbidity including type 2 diabetes and lose 15–20 years of life due to cardiovascular diseases, with early accrual of cardiometabolic disease. In this study, thirty overweight or obese (Body Mass Index (BMI) > 25) participants under 40 years old with schizophrenia spectrum disorders and early comorbid prediabetes or type 2 diabetes receiving antipsychotic medications were randomized, in a double-blind fashion, to metformin 1500 mg/day or placebo (2:1 ratio; *n* = 21 metformin and *n* = 9 placebo) for 4 months. The primary outcome measures were improvements in glucose homeostasis (HbA1c, fasting glucose) and insulin resistance (Matsuda index—derived from oral glucose tolerance tests and homeostatic model of insulin resistance (HOMA-IR)). Secondary outcome measures included changes in weight, MRI measures of fat mass and distribution, symptom severity, cognition, and hippocampal volume. Twenty-two patients (*n* = 14 metformin; *n* = 8 placebo) completed the trial. The metformin group had a significant decrease over time in the HOMA-IR (*p* = 0.043) and fasting blood glucose (*p* = 0.007) vs. placebo. There were no differences between treatment groups in the Matsuda index, HbA1c, which could suggest liver-specific effects of metformin. There were no between group differences in other secondary outcome measures, while weight loss in the metformin arm correlated significantly with decreases in subcutaneous, but not visceral or hepatic adipose tissue. Our results show that metformin improved dysglycemia and insulin sensitivity, independent of weight loss, in a young population with prediabetes/diabetes and psychosis spectrum illness, that is at extremely high risk of early cardiovascular mortality. Trial Registration: This protocol was registered with clinicaltrials.gov (NCT02167620).

## Introduction

Patients with schizophrenia have exceedingly high rates of metabolic comorbidity including obesity, dyslipidemia, and type 2 diabetes, all of which contribute to the high rates of mortality and morbidity seen among this patient population. The prevalence of type 2 diabetes in the schizophrenia population is 3–9 fold higher than the general population^1,2^, and patients with schizophrenia die on average 15–20 years earlier from cardiovascular disease^3^. This high comorbidity is due to a combination of endogenous (i.e. genetics) and exogenous factors (i.e. lifestyle factors, reduced access to physical care, and medications). Among these factors, antipsychotic drugs, the cornerstone of schizophrenia treatment, contribute significantly to this risk^3^. Young patients in the earliest stages of their illness are especially vulnerable to antipsychotic-induced metabolic dysfunction as reflected in rates of glucose intolerance (55%) or impaired fasting glucose (21%) observed as early as within the first year of treatment^4,5^. Both younger age and lack of previous exposure to antipsychotic medications represent risk factors for the development of antipsychotic-induced metabolic adverse effects^6^. Furthermore, metabolic dysfunction, including glucose dysregulation, occurs rapidly after exposure to antipsychotics^7,8^. These metabolic complications have wide-ranging detrimental effects on numerous domains including cognitive performance^9^, medication compliance^10^, self-esteem, and quality of life^11^.

Unfortunately, rates of non-treatment for these medical conditions are high in schizophrenia (>30% for type 2 diabetes)^12^. Furthermore, patients with schizophrenia are typically systematically excluded from trials investigating antidiabetic agents, while studies investigating treatments for weight gain in schizophrenia typically exclude patients with type 2 diabetes resulting in a lack of evidence to guide treatment. This is important, as patients with schizophrenia may not share common mechanisms to insulin resistance associated with obesity and type 2 diabetes in the general population. To this point, schizophrenia itself represents a biological risk factor for type 2 diabetes^13^ while antipsychotics have been shown to impact pathways of glucose metabolism independently of weight gain^14,15^. To the best of our knowledge, only two studies in schizophrenia have examined antidiabetic agents (rosiglitazone and glucagon-like-peptide-1 receptor agonists) in patients with insulin resistance or impaired fasting blood glucose^16,17^. In animal models, commonly used antidiabetic agents only partly reverse antipsychotic-induced disruptions in glucose homeostasis^18,19^, highlighting the importance of specifically conducting studies in schizophrenia patients with comorbid dysglycemia.

Metformin is the first-line pharmacologic treatment for type 2 diabetes, and represents the most widely prescribed drug worldwide for this indication^20^. The major mechanism of action for metformin involves suppression of hepatic glucose production (via AMPK activation), in addition to the action on the gut to increase glucose utilization^20^. In patients with schizophrenia, metformin has been widely studied “off-label” for antipsychotic-related weight gain, and has the most evidence supporting efficacy and safety in this role^21^. It is now recommended in the most recent Canadian Obesity Guidelines in conjunction with lifestyle modification for antipsychotic-induced weight gain^22^. It has been shown in some studies to improve insulin resistance as assessed by the homeostatic model of insulin resistance (HOMA-IR), a static/surrogate measure of insulin sensitivity. However, most studies exclude patients with overt glucose dysregulation. It is unclear whether patients with schizophrenia spectrum disorders and prediabetes or type 2 diabetes should be treated with metformin first line, or if they might require combination strategies and/or more intensive intervention approaches.

In this pilot study, we examined whether metformin is efficacious in reversing glucose dysregulation in a young population of patients (ages 17–45) within 5 years of a DSM-5 diagnosis of schizophrenia, schizoaffective disorder, or bipolar disorder, or under the age of 40 (regardless of illness duration) and a diagnosis of type 2 diabetes or prediabetes. We hypothesized that the addition of metformin would decrease HbA1c and improve calculated indices of insulin sensitivity (including the Matsuda index^23^, and HOMA-IR), reduce glucose excursion during oral glucose tolerance tests (OGTTs), and increase the insulin secretion sensitivity index-2 (ISSI-2)^24^ vs. placebo. Secondary outcomes of interest included weight, adiposity-related measures, including proportion losing >5% body weight. Given the close association between cardiovascular risk, visceral fat, and hepatic fat, we also measured these outcomes using state-of-the-art magnetic resonance imaging (MRI) procedures. Furthermore, given recent evidence linking glucose dysregulation, cognitive impairment, and changes in hippocampal volume^25^, we also examined the cognitive performance and hippocampal volume (using MRI) as exploratory outcomes.

## Materials and Methods

### Participants

Clinically stable, overweight (body mass index (BMI) > 25) patients (ages 17–45) within 5 years of a DSM-5 diagnosis of schizophrenia, schizoaffective disorder, or bipolar disorder, or under the age of 40 (regardless of illness duration), with comorbid prediabetes or type 2 diabetes (American Diabetes Association criteria)^26,27^ were approached for the study between June 2014 and March 2018 at the Centre for Addiction and Mental Health (CAMH) in Toronto, Canada. Eligibility required a stable antipsychotic dose for three months prior to study enrollment. The protocol was approved by the CAMH Research Ethics Board, and was registered with clinicaltrials.gov (NCT02167620) before participant enrolment. Informed consent was obtained from all patients prior to beginning the study. Patients were excluded if they had a comorbid psychiatric disorder, other than nicotine or cannabis dependence, type 1 diabetes (confirmed using fasting bloodwork results), liver or renal dysfunction (AST > 37, albumin <34, ALP > 116, GGT > 55), positive drug urine screen (other than cannabis or nicotine), HbA1c > 9.5% or symptomatic hyperglycemia with metabolic decompensation, previously had received metformin and reported lack of tolerability/efficacy, the addition of new hypoglycaemic or lipid-lowering medication within 3 months of study entry, and switching antipsychotic medications within 3 months of study entry. Female participants with a positive pregnancy test were also excluded.

### Study design

A double-blind design was used, with participants randomized in a 2:1 ratio to 16 weeks of treatment with immediate-release metformin or placebo dispensed by the CAMH research pharmacy in identical capsules. Randomization was determined using random numbers tables using random block sizes by the CAMH research pharmacy. Study staff and participants remained blinded until study completion. All participants, regardless of randomization status, also received a lifestyle counseling focusing on diet and exercise by a registered dietician. Metformin (or matching placebo) was initiated at 500 mg OD, increased to 500 mg BID after 7 days and, if tolerated, at day 14, increased to 750 mg BID. Patients not tolerating a dose increase were maintained on the highest tolerated dose (or tablet placebo equivalent). Participants were required to bring back empty blister packs to monitor adherence.

### Outcomes

Participants were assessed bi-weekly for anthropometric measures (weight, waist circumference, body mass index (BMI)) and side effects using the Udvalg for Klinske Undersogelser (UKU) drug side effect scale^28^. The Brief Psychiatric Rating Scale (BPRS)^29^, Clinical Global Impression (CGI) Scale^29^, and Calgary Depression Scale (CDSS)^30^ were used to assess clinical symptoms while the Brief Assessment of Cognition in Schizophrenia (BACS) was used to measure cognitive function at baseline and endpoint. Blinded raters performed all assessments. An OGTT and an MRI scan of the brain and abdomen were performed before and after the 16-week treatment period. The primary outcome measures were improvements in glucose homeostasis (HbA1c, fasting glucose) and insulin resistance (Matsuda index—derived from oral glucose tolerance tests and homeostatic model of insulin resistance (HOMA-IR)). Secondary outcome measures included changes in weight, MRI measures of fat mass and distribution, symptom severity, cognition, and hippocampal volume.

### Oral glucose tolerance test (OGTT) protocol

The OGTT involved administration of a standard glucose drink (75 g) after overnight fasting and blood samples were obtained at 0, 60, and 120 min for measurements of insulin, c-peptide, and glucose. Whole-body insulin sensitivity was calculated based on the description by Matsuda^23^, which has a high degree of correlation with gold-standard euglycemic-hyperinsulinemic clamp derived values^23,31,32^. ß-cell function was assessed using the Insulin Secretion-Sensitivity Index-2 (ISSI-2), an OGTT-derived measure analogous to the disposition index derived from the intravenous glucose tolerance test^24^. We also measured the area under the curve (AUC) for glucose.

### MRI scanning protocol

All patients underwent abdominal MRI scans in a Siemens Skyra 3T dedicated research MRI scanner (Siemens Healthcare, Germany) at St. Michael’s Hospital. A T1-weighted image (TR = 2300 ms, Non-selective TI = 1100 ms, echo time=3.55 ms, 176 slices, 0.9 mm isotropic) of the brain was acquired to measure hippocampal volume. For visceral and abdominal fat quantification a chemical-shift-based water-fat pulse sequence (Dixon) based on a 3D spoiled gradient echo with multi-peak spectral modeling of fat and correction for T2* variations was used^33^. Three axial 5 mm contiguous slices were acquired in a single breath hold (10-15 s), at L4-L5 level (estimated in plane resolution 1.5-2.5 mm; TR 9- 11 ms; TE1 = 0.57 ms, TE2 = 1.7 ms and TE3 = 2.8 ms allowing, at 3T, approximation of the following phase relations between water and fat: 90 deg, 270 deg, and 450 deg). Liver fat fraction averages were obtained via a Multi Echo T2 corrected Single Voxel Spectroscopy (HISTO) specifically developed by Siemens for liver fat quantification. This modified single voxel STEAM sequence is performed in one breath hold, with a total acquisition time of 15 s and integrated post-processing. Three measurements were made in the right hepatic lobe of each patient (anterior, mid, and posterior) avoiding major vascular structures.

### Power calculation

As this was a pilot study investigating the efficacy of metformin in a population that has not been systematically studied, a sample size calculation was not performed. Our final sample size was powered to identify moderate to large effect size changes in the primary outcome of interest.

### Statistical analyses

All participants who were randomized and had received at least 1 dose of metformin or placebo were included in the analyses. Initial descriptive analysis was conducted to describe the profile of the sample and to investigate differences between intervention groups at baseline on main demographics and clinic measures. Hippocampal volumes were measured using the MAGeT-Brain (Multiple Automatically Generated Templates) algorithm, a method modeling population variability inherent in any dataset to provide accurate and robust volume estimates. This method, developed in collaboration with CAMH, has recently been compared to manual segmentations in younger (often first episode) schizophrenia populations and is shown to be superior to commonly used FreeSurfer and FSL FIRST methods^34^.

For abdominal fat measurement, images were exported to 3D slicer software (Version 4.6.2) for supervised segmentation, using different tools such as selective thresholding and manual contouring, to estimate subcutaneous (SAT) and visceral fat (VAT)^35^. SAT and VAT for each subject were measured by segmenting the appropriate fat pixels on each of the three slices and then measuring the total volume of the segmented pixels in mL. The liver fat percentage was obtained by using the vendor-provided inline calculations. An arithmetic mean liver fat value to be used in the subsequent analysis was calculated for each patient using the three measured values.

A mixed model analysis was used with HbA1c and measures of insulin sensitivity as the primary outcome measures; time (study week), group (metformin vs. placebo) and the interaction between group and time, were included as predictor variables. Pre-identified covariates (i.e. baseline BMI) were included in the model. A similar approach was used for the secondary/exploratory outcome measures.

## Results

### Trial population and baseline characteristics

**Fig. 1:**
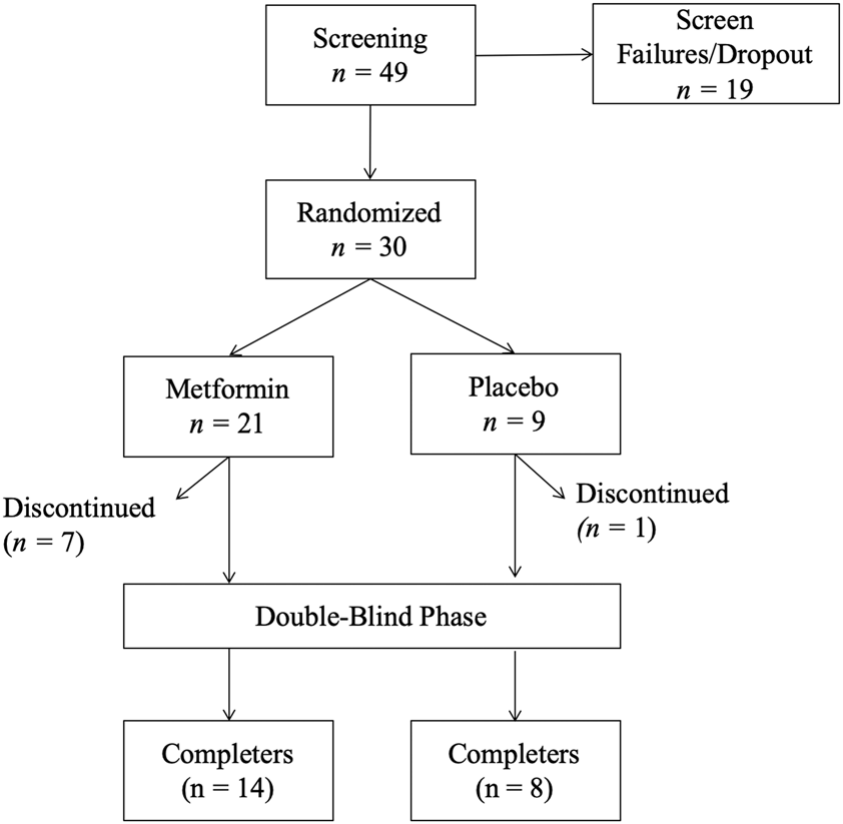
Flowchart of study participants. All participants who were randomized and had received at least 1 dose of metformin or placebo were included in the analyses.

Of 49 eligible participants, 30 participants were randomized to receive metformin or placebo in the study and 22 completed the study; 14 in the metformin and 8 in the placebo arm (Fig. 1). Randomization resulted in balanced baseline clinical and demographics characteristics between arms (Table 1). Number and reasons for dropping out did not differ between groups (eTable 1 in the Supplement). Concomitant medications as assessed at baseline are shown in Table 1. The participants who did not complete the study had higher mean weight and waist circumference than study completers at baseline but did not differ with respect to primary outcomes or other essential clinical and demographic characteristics at baseline (eTable 2 in the Supplement). OGTT data were available for 19 metformin and 9 placebo arm participants at baseline and 13 metformin and 8 placebo arm participants at follow-up while imaging data were available for 18 metformin and 7 placebo arm participants at baseline and 12 metformin and 7 placebo arm participants at follow-up.

**Table 1:** Baseline characteristics of the study participants.

Characteristic	Treatment group		
	Metformin *N* = 21	Placebo *N* = 9	*P* Value
**Sociodemographic**			
Age, mean (SD), y	31.4 (6.51)	32.2 (6.14)	0.758
Male, No. (%)	12 (57.1)	2 (22.2)	.0740
Diagnosis, No. (%)			
Schizophrenia	12 (57.1)	3 (33.3)	
Psychosis	0	1 (11.1)	
Schizoaffective disorder	1 (4.8)	2 (22.2)	
Multiple diagnosis	6 (28.6)	2 (22.2)	
Paranoid schizophrenia	0	1 (11.1)	
Bipolar disorder	2 (9.5)		
Age of illness onset, mean (SD), y	23.8 (7.66)	21.3 (4.90)	
Duration of diagnosis, mean (SD), y	7.53 (6.16)	10.94 (7.58)	
Treatment, No. (%)			
*High weight gain potential*			
Clozapine	5 (23.8)	1 (11.1)	
Olanzapine	2 (9.52)		
*Moderate weight gain potential*			
Paliperidone		1 (11.1)	
Quetiapine	3 (14.3)		
Risperidone	2 (9.52)		
Risperidone injectable		1 (11.1)	
Zuclopenthixol	1 (4.76)		
Paliperidone palmite	1 (4.76)		
Flupentixol	1 (4.76)		
*Low weight gain potential*			
Aripiprazole	1 (4.76)	4 (44.4)	
Perphenazine	1 (4.76)		
Ziprasidone	1 (4.76)		
*Polytherapy*	3 (14.3)	2 (22.2)	
Dose, mean (SD), in CPZ equivalents			
Paliperidone		300 (NA)	
Quetiapine oral	341.88 (139.54)	227.92 (NA)	
Risperidone injectable		200 (NA)	
Aripiprazole	313.63 (88.69)	267.21 (78.91)	
Perphenazine	175.88 (NA)		
Risperidone	221.65 (95.47)		
Zuclopenthixol	600 (NA)		
Clozapine	270.23 (99.23)	228.22 (145.64)	
Ziprasidone	159.68 (NA)		
Paliperidone injectable	156 (NA)		
Flupentixol oral	33.33 (NA)		
Flupentixol injectable	200 (NA)		
Olanzapine	219.54 (54.23)		
Clinical characteristics, mean (SD)			
Body weight, kg	105.3 (29.2)	114.8 (27.1)	0.409
Waist circumference, cm	114.6 (19.3)	127. 1 (20.3)	0.990
BMI	38.8 (15.1)	42.4 (9.86)	0.525
Systolic blood pressure, mm Hg	122.8 (13.1)	117.8 (16.3)	0.381
Diastolic blood pressure, mm Hg	76.7 (9.18)	83.1 (18.6)	0.349
Prediabetes criteria, No. (%)			
Elevated fasting plasma glucose level	10 (47.6)	3 (33.3)	0.469
Elevated glycated hemoglobin level	13 (61.9)	5 (55.6)	0.745
Impaired glucose tolerance	8 (38.1)	4 (44.4)	0.745
>1 Criterion of prediabetes	11 (52.4)	4 (44.4)	0.690
Diabetes criteria, No. (%)			
Elevated fasting plasma glucose level	3 (14.3)	1 (11.1)	0.815
Elevated glycated hemoglobin level	0	2 (22.2)	–
Impaired glucose tolerance	5 (23.8)	4 (44.4)	0.258
>1 Criterion of Diabetes	2 (9.52)	1 (11.1)	0.894
Glucose metabolism			
Glycated hemoglobin level, mean (SD) %	5.79 (.379)	6.30 (1.39)	0.309
Fasting plasma glucose level, mean (SD), ng/mL	5.87 (.748)	5.74 (.8819)	0.689
Fasting C-peptide secretion, mean (SD), ng/mL	1299.3 (485.8)	1395.3 (495.5)	0.631
Fasting glucagon secretion, mean (SD), pg/mL	9.03 (2.90)	12.1 (5.93)	0.071
Insulin resistance (HOMA-IR), mean (SD)	4.94 (3.15)	3.72 (1.35)	0.275
Beta cell function (ISSI-2), mean (SD)	136.1 (45.6)	118.8 (72.3)	0.448
Insulin sensitivity (Matsuda Index), mean (SD)	2.03 (1.34)	1.60 (1.19)	0.421
2-h, 75-g OGTT finding, mean (SD) mg/dL	9.09 (2.86)	11.6 (5.70)	0.110
Body composition^a^			
Visceral fat, mean (SD), cm^3^	206.8 (106.5)	242.5 (146.8)	0.475
Subcutaneous to visceral fat ratio, mean (SD)	3.72 (1.31)	5.08 (3.27)	0.135
Liver Fat (SD), %	33.14 (26.4)	39.9 (33.03)	0.639
Brain imaging^b^			
Right hippocampus gray matter volume (SD) mm^3^	1980.61 (352.4)	1962.40 (282.17)	0.904
Left hippocampus gray matter volume (SD) mm^3^	2003.77 (315.29)	2035.28 (336.07)	0.827
Cholesterol level, mean (SD), mmol/L			
Total	5.05 (.825)	4.90 (.510)	0.623
LDL	3.20 (.843)	3.04 (.532)	0.638
HDL	1.12 (.266)	1.12 (.208)	0.981
Triglycerides	1.62 (.718)	1.62 (.927)	0.998
Rating scales			
CGI-S	3.62 (1.24)	3.11 (.601)	0.144
GAF	51.7 (16.7)	52.7 (12.2)	0.877
BPRS	32.0 (9.39)	28.6 (5.36)	0.314
BACS composite t score	26.0 (21.0)	33.2 (12.8)	0.346
BACS Verbal Memory t score	32.3 (18.9)	32.9 (10.4)	0.934

*BMI* body mass index (calculated as weight in kilograms divided by height in meters squared), *BPRS* Brief Psychiatric Rating Scale, *BACS* Brief Assessment of Cognition in Schizophrenia, *CGI* Clinical Global Impressions Scale severity score, *GAF* Global Assessment of Functioning scale, *HDL* high-density lipoprotein, *LDL* low density lipoprotein, *SD* standard deviation.

^a^Body composition outcomes were available for 18 participants in the metformin arm and 9 in the placebo arm.

^b^Brain imaging outcomes were available for 18 participants in the metformin arm and 7 in the placebo arm at baseline.

### Glycemic control

**Fig. 2:**
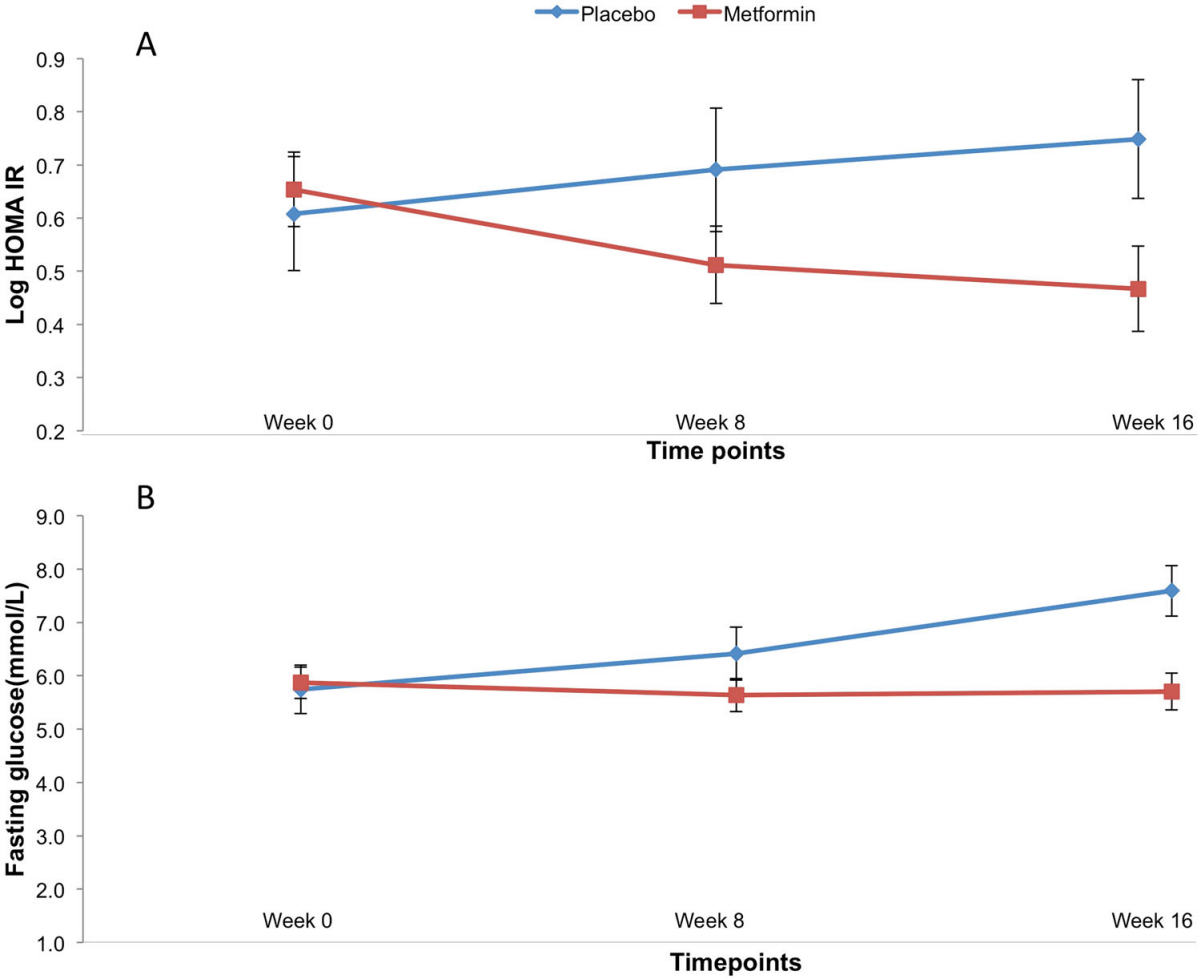
Change in insulin sensitivity (HOMA-IR) (A) and fasting glucose levels (B) with metformin compared to placebo at baseline, 8 weeks, and 16 weeks of treatment. Error bars represent ± 1 S.E.

The metformin arm demonstrated improvement in insulin sensitivity as measured using HOMA-IR after the 16-week treatment period (*F* = 3.3, *p* = 0.043) (Fig. 2A, Table 2). There was no significant difference between the treatment arms with respect to HbA1c (*F* = 0.7; *p* = 0.5), Matsuda Index (*F* = 0.9, *p* = 0.4), or ISSI-2 (*F* = 0.4, *p* = 0.9). The difference in HOMA-IR was driven by difference in fasting glucose levels (*F* = 5.5, *p* = 0.007) (Fig. 2B, Table 2) in the metformin arm but not the placebo arm (Table 2). There was no difference in glucose tolerance (glucose excursion during 2-h OGTT) between groups. Controlling for baseline BMI did not change the findings.

**Table 2:** Change in outcome measures from Baseline to Week 16.

Characteristic	Metformin treatment group *N* = 14	Placebo treatment group *N* = 8	Estimated treatment difference, Metformin vs Placebo (95% CI)	*p* value
*Clinical, mean (SE)*				
Body weight, kg	−3.57 (1.19)	−0.614 (1.73)	−2.95 (−7.10 to 1.18)	0.161
Waist circumference, cm	−3.25 (1.47)	−6.44 (2.05)	3.18 (−1.80 to 8.17)	0.209
BMI	−1.11 (1.31)	−0.36 (1.79)	−0.75 (−5.13 to 3.63)	0.736
Systolic blood pressure mm Hg	−6.32 (4.18)	2.37 (5.87)	−8.700 (−23.37 to 5.97)	0.236
Diastolic blood pressure, mm Hg	1.29 (4.12)	−9.87 (5.82)	11.16 (−3.08 to 25.40)	0.122
*Glucose metabolism*				
Glycated hemoglobin level, %	−0.05 (0.08)	0.085 (0.1)	−0.1430 (−0.42 to 0.14)	0.317
Fasting plasma glucose level	−0.16 (0.35)	1.84 (0.49)	−2.01 (−3.23 to -0.79)	0.007
Fasting C-peptide level	−10.12 (137.30)	−46.55 (183.86)	36.43 (−438.72 to 511.58)	0.875
Log Insulin resistance (HOMA-IR)	−0.12 (0.08)	0.21 (0.12)	−0.33 (−0.63 to -0.037)	0.043
Beta cell function	11.99 (7.68)	9.57 (9.85)	2.42 (−23.70 to 28.55)	0.848
Insulin sensitivity (Matsuda)	0.83 (4.34)	10.93 (5.86)	−10.10 (−25.09 to 4.89)	0.178
Fasting plasma insulin level	−12.8 (23.5)	67.01 (33.3)	−79.8 (−161.6 to 1.9)	0.056
2-hr, 75-g OGTT value	−0.63 (0.52)	−1.12 (0.67)	0.48 (−1.30 to 2.27)	0.577
*Body composition*^1^				
Visceral fat, mean (SD), cm^3^	35.2 (28.3)	−34.1 (34.3)	69.3 (−24.3 to 16.3)	0.137
Subcutaneous fat, mean (SD), cm^3^	−9.46 (27.0)	20.4 (32.4)	−29.9 (−12.0 to 60.0)	0.489
Subcutaneous to visceral fat ratio, mean (SD)	−0.20 (0.18)	0.33 (0.22)	-0.54 (−1.17 to 0.07)	0.080
Liver Fat (SD), %	1.042 (4.44)	−0.603 (4.458)	1.645 (−12.904 to 16.196)	0.800
*Brain imaging*^2^				
Right hippocampus gray matter volume (SD) mm^3^	24.489 (23.24)	−2.824 (30.508)	27.31 (−53.593 to 108.220)	0.486
Left hippocampus gray matter volume (SD) mm^3^	15.884 (17.020)	17.402 (22.313)	−1.518 (−60.722 to 57.684)	0.957
*Cholesterol level (mmol/L)*				
Total, mean (SD)	−0.466 (0.23)	−0.23 (0.33)	−0.23 (−1.07 to 0.61)	0.577
LDL, mean (SD)	−0.34 (0.20)	−0.15 (0.28)	−0.19 (−0.93 to 0.54)	0.591
HDL	−0.090 (0.05)	−0.02 (0.07)	−0.06 (−0.26 to 0.13)	0.521
Fasting triglyceride level	0.004 (0.16)	−0.18 (0.23)	0.18 (−0.40 to 0.77)	0.523
*Rating scales*				
CGI	−0.10 (0.20)	0.25 (0.28)	−0.35 (−1.08 to 0.36)	0.317
GAF	2.60 (2.75)	1.88 (3.75)	0.71 (−8.96 to 10.39)	0.879
BPRS	−2.66 (1.60)	−0.31 (2.27)	−2.35 (−7.94 to 3.24)	0.403
BACS composite t score	2.23 (1.74)	0.97 (2.30)	1.26 (−4.76 to 7.29)	0.667
BACS Verbal Memory t score	4.48 (7.07)	20.51 (10.68)	−16.02 (−42.35 to 10.30)	0.22

^a^Body composition outcomes for both time points were available for 10 participants in the metformin arm and 7 in the placebo arm.

^b^Brain imaging outcomes for both time points were available for 12 participants in the metformin arm and 7 in the placebo arm.

### Body weight and metabolic variables

No change was observed in any other anthropometric or lipid parameters with metformin treatment (all *p* > 0.05, Table 2**)**.

### Exploratory outcomes

**Fig. 3:**
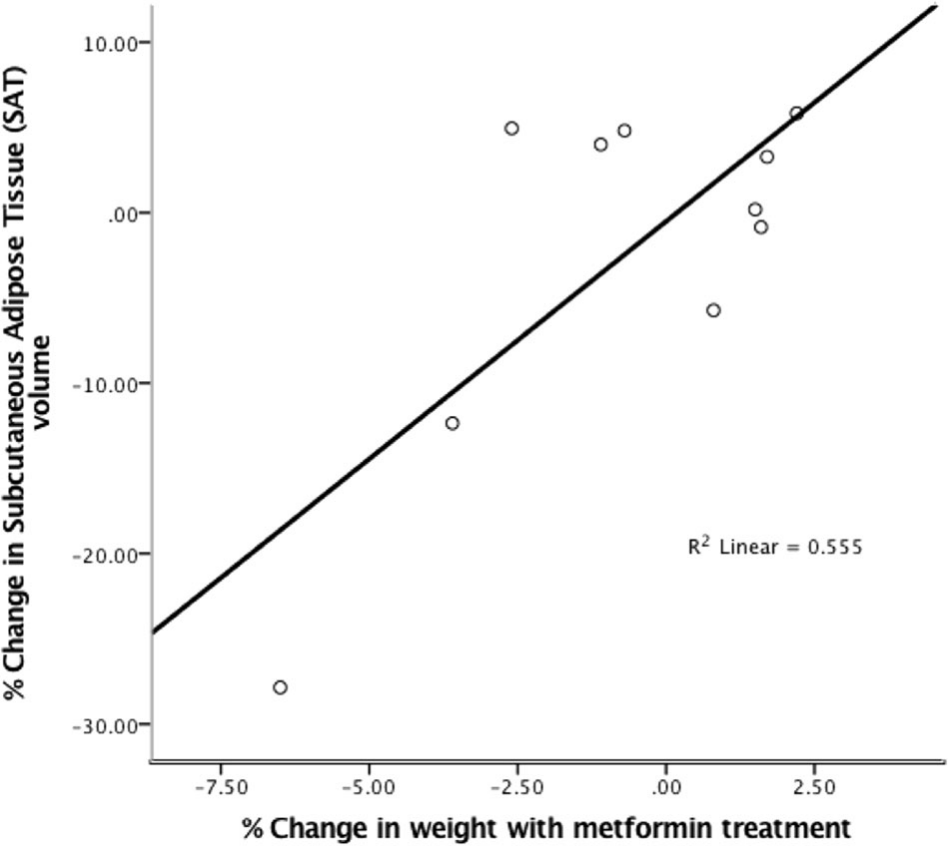
Correlation between percentage change in weight and subcutaneous adipose tissue (SAT) volume with metformin treatment.

There were no differences between treatment arms with respect to change in visceral, subcutaneous, or hepatic adiposity over 16 weeks; percentage of participants with >5% weight loss (16.67% vs 12.5%; *p* = 0.8). Similarly, no between group differences were noted in cognitive performance; psychopathology severity; or, hippocampal volume (all *p* > 0.05, Table 2**)**. With metformin, percentage decrease in weight correlated with decrease in subcutaneous but not visceral adipose tissue (*r* = 0.8, *p* = 0.006, Fig. 3). Exploratory correlations between change in metabolic indices and change in clinical and cognitive parameters were non-significant.

### Adverse effects

No statistically significant difference in the frequency of side effects was noted; gastrointestinal side effects were the most common in either group (eTable 3 in the Supplement). One metformin subject died for reasons unrelated to study participation as illustrated by autopsy.

## Discussion

In patients under age 40 with comorbid schizophrenia spectrum disorders and type 2 diabetes or prediabetes, metformin treatment over 16 weeks resulted in lower HOMA-IR and fasting glucose levels. No statistically significant changes were noted in other outcomes. Metformin was well-tolerated, with no difference in adverse effects between groups.

Metformin has previously been shown to increase weight loss and improve insulin sensitivity (as measured by HOMA-IR) in patients with schizophrenia^21^. Improvement in HOMA-IR with metformin was replicated in this study, but weight loss effects were not significant. Notably, patients in this study had overt glucose dysregulation, a sample routinely excluded in studies examining weight-loss interventions in schizophrenia. Interestingly, a meta-analysis of metformin studies in schizophrenia noted greater efficacy early in the illness;^36^ possibly weight loss effects are blunted once patients develop prediabetes/diabetes.

Of note is the divergence of metformin’s effect on measures derived from fasting and post-glucose load derived parameters of insulin sensitivity. Metformin improved fasting blood glucose, and HOMA-IR, but not the insulin sensitivity index derived from the OGTT (i.e. Matsuda index). The small sample size precludes firm conclusions, but several factors warrant comment. HOMA-IR is often considered a measure of hepatic insulin resistance, while Matsuda represents a whole-body measure of insulin sensitivity and depends on not only hepatic but also skeletal glucose disposition. Metformin’s primary effect is thought to occur through reductions in hepatic glucose production with overall reductions in insulin resistance largely attributable to hepatic effects. Metformin has been shown to modulate AMPK (AMP-activated protein kinase), a key regulator of energy homeostasis in the liver^37^ as well as the duodenum to reduce hepatic glucose production^38^. It can also cross the blood-brain barrier and act on the hypothalamus. In diabetic rats, following oral administration, metformin was found in cerebrospinal fluid and reduced food intake by reducing the expression of orexigenic peptides^39^. It also normalized intrahypothlamic levels of leptin and insulin, as well AMPK activity, translating to improvement in liver function in obese agouti mice^40^. Interestingly, in rodents we have demonstrated olanzapine induced whole-body insulin resistance, with metformin reversing hepatic, but not peripheral, insulin resistance^18^. This leads to the interesting possibility that metformin preferentially acts on hepatic and non-hepatic targets to reduce hepatic resistance but does not improve insulin sensitivity in other important targets such as skeletal muscle or adipose tissue in the context of antipsychotic-induced dysglycemia. Failure to effect HbA1c aligns with the well-established positive relationship between high baseline HbA1c and magnitude of HbA1c change with interventions^41^. The lower baseline mean value of HBA1c found in this study (mean = 5.9%) may help explain absence of group differences. Furthermore, the 16-week period may not have been long enough to capture changes in HbA1c and further the lack of metformin’s efficacy on non-hepatic targets might have blunted its effect on HbA1c.

Interestingly, weight loss in the metformin arm was associated with decrease in subcutaneous fat but not visceral fat. Visceral adipose tissue might be of greater importance in preventing cardiovascular disease, as it is a better predictor of metabolic risk than BMI^42^. Hence, metformin’s apparently selective action on subcutaneous fat but not visceral fat needs to be investigated further in future studies to understand its impact on overall metabolic risk in patients on antipsychotics.

No significant differences were also noted for metabolic (i.e. lipid), exploratory imaging (adiposity, hippocampal volumes), or cognitive parameters that could be reflective of the small sample size. Furthermore, due to the small sample sizes, we were unable to explore differences between key subgroups (e.g. pre-diabetic vs. diabetic and overweight vs. obese), which could further assist in study efficacy. Nevertheless, we offer novel data on metformin’s utility in a young, under-researched population of patients with schizophrenia spectrum disorders who are at extremely high risk for future CVD. In addition to this, we did not assess for Maturity Onset Diabetes of the Young. Our findings support the use of metformin in this patient group as one arm of a broader strategy to address dysglycemia, but raise the issue of treatment initiation before the development of prediabetes/T2D to obtain maximum benefits. The results of this study will be used to design a larger longitudinal study that will examine the role of metformin in a preventative role before the onset of prediabetes/diabetes to address the early metabolic risk accrual cumulating in premature CV mortality in those suffering from severe mental illness.

## Supplementary information

Supplementary Tables

CONSORT Checklist
