# Supplementary material for: Metformin for early comorbid glucose dysregulation and schizophrenia spectrum disorders: a pilot double-blind randomized clinical trial
Source: Transl Psychiatry. 2021 Apr 14;11:219. doi: 10.1038/s41398-021-01338-2 (PMC8046796; doi:10.1038/s41398-021-01338-2)
Supplement: Supplementary file 1 — Supplementary Tables [file 41398_2021_1338_MOESM1_ESM.docx]

**Supplementary tables**

**eTable 1: Study discontinuation during double-blind treatment**

| Reasons1 | No. of Dropouts | | p |
| --- | --- | --- | --- |
|  | Metformin (n=21) | Placebo (n=9) |  |
| Adverse events | 2 | 0 |  |
| Change in medication | 1 | 1 |  |
| Death | 1 | 0 |  |
| Lack of efficacy | 1 | 0 |  |
| Lost to follow-up | 1 | 0 |  |
| Pregnancy | 1 | 0 |  |
| Unspecified/Other | 1 | 0 |  |
|  |  |  |  |
| Total no. (%) of participants who discontinued | 7 (33.3) | 1 (11.1) | 0.4 |
| Mean no. of weeks  in study (SD) | 5.82 (3.24) | 3 (0) | n/a |
|  |  |  |  |
| ^1^A patient may have more than one reason for discontinuing | | |  |

**eTable 2: Comparison between participants who completed or dropped out during double-blind treatment**

|  | Completion status | | |
| --- | --- | --- | --- |
| Characteristic | Completer  N=22 | Dropout  N=8 | P Value |
| Sociodemographic |  |  |  |
| Age, mean (SD), y | 32.0 (7.03) | 30.75 (3.88) | .545 |
| Male, No. (%) | 12 (55) | 2 (25) | .155 |
| Diagnosis, No. (%) |  |  |  |
| Schizophrenia | 11 (50) | 4 (50) |  |
| Bipolar Disorder | 1 (4.5) | 1 (12.5) |  |
| Multiple Diagnoses | 5 (22.7) | 3 (37.5) |  |
| Psychosis | 1 (4.5) |  |  |
| Schizoaffective Disorder | 3 (13.6) |  |  |
| Paranoid Schizophrenia | 1 (4.5) |  |  |
| Age of illness onset, mean (SD), y | 23.73 (7.28) | 21.13 (5.99) | .374 |
| Duration of diagnosis, mean (SD), y | 8.21 (6.52) | 9.5 (7.46) | .648 |
| Clinical characteristics, mean (SD) |  |  |  |
| Body weight, kg | 101.12 (24.4) | 127.4 (31.3) | .022 |
| Waist circumference, cm | 113.3 (17.3) | 132.0 (22.3) | .022 |
| BMI | 37.9 (14.3) | 45.23 (10.6) | .203 |
| Systolic blood pressure, mm Hg | 120.4 (13.6) | 124 (15.7) | .540 |
| Diastolic blood pressure, mm   Hg | 77.0 (12.2) | 82.9 (14.1) | .271 |
| Glucose metabolism |  |  |  |
| Glycated hemoglobin level,   mean (SD) % | 6.0 (0.94) | 5.9 (0.42) | .867 |
| Fasting plasma glucose level,   mean (SD), ng/mL | 5.87 (.778) | 5.73 (.817) | .653 |
| Fasting C-peptide secretion,   mean (SD), ng/mL | 1337.0 (543.2) | 1305.2 (115) | .801 |
| Insulin resistance (HOMA-IR), mean (SD) | 4.59 (3.05) | 4.53 (1.96) | .957 |
| Beta cell function (ISSI-2), mean (SD) | 129.1 (57.1) | 135.7 (49.8) | .787 |
| Insulin sensitivity (Matsuda Index), mean (SD) | 1.93 (1.42) | 1.73 (.703) | .744 |
| 2-h, 75-g OGTT finding, mean   (SD) mg/dL | 10.4 (4.29) | 8.43 (2.82) | .248 |
| Body composition |  |  |  |
| Visceral fat, mean (SD), cm3 | 227.9 (133.2) | 192.4 (69.7) | .510 |
| Subcutaneous to visceral fat   ratio, mean (SD) | 3.56 (1.16) | 5.90 (3.47) | .128 |
| Cholesterol level, mean (SD), mmol/L |  |  |  |
| Total | 5.1 (.79) | 4.78 (0.6) | .310 |
| LDL | 3.3 (.80) | 2.9 (.61) | .198 |
| HDL | 1.07 (.21) | 1.25 (.31) | .089 |
| Triglycerides | 1.67 (.85) | 1.49(.48) | .586 |
| Rating Scales |  |  |  |
| CGI-Se | 3.27 (.883) | 4 (1.51) | .113 |
| GAF | 51.95 (14.1) | 52.17 (20.8) | .977 |
| BPRS | 29.2 (6.5) | 35.9 (11.4) | .153 |
| BACS composite t score | 28.70 (18.06) | 26.63 (22.62) | .798 |
| BACS Verbal Memory t score | 32.30 (18.57) | 33.33 (10.26) | .873 |

**eTable 3: Comparison of adverse events frequency between study arms**

| No. (%) of Participants | | | |
| --- | --- | --- | --- |
| Adverse Event or Reaction | Metformin arm (N=21) | Placebo arm (N=9) | P value |
| Autonomic |  |  |  |
| Nausea | 9 (42.9) | 5 (55.6) | 0.523 |
| Diarrhea | 8 (38.1) | 7 (77.8) | 0.046 |
| Constipation | 4 (19.0) | 4 (44.4) | 0.149 |
| Vomiting | 3 (14.3) | 2 (22.2) | 0.592 |
| Abdominal pain | 2 (9.52) | 1 (11.1) | 0.894 |
| Dysgeusia | 2 (9.52) |  |  |
| Decreased appetite | 2 (9.52) | 2 (22.2) | 0.348 |
| Functional dyspepsia | 1 (4.8) | 2 (22.2) | 0.144 |
| Acid reflux | 2 (9.52) |  |  |
| Abdominal bloating | 3 (14.3) |  |  |
| Increased appetite | 1 (4.8) | 1 (11.1) | .523 |
| Xerostomia | 2 (9.52) |  |  |
| Psychiatric related |  |  |  |
| Increase in psychosis | 1 (4.8) |  |  |
| Depression |  | 1 (11.1) |  |
| Irritated/bad mood | 1 (4.8) |  |  |
| Psychic |  |  |  |
| Fatigue | 1 (4.8) | 2 (22.2) | 0.144 |
| Headache | 1 (4.8) | 1 (11.1) | 0.523 |
| Dizziness | 3 (14.3) | 3 (33.3) | 0.231 |
| Light headedness | 1 (4.8) | 1 (11.1) | 0.523 |
| Difficulty concentrating |  | 1 (11.1) |  |
| Neurologic |  |  |  |
| Myalgia (muscle pain) | 1 (4.8) |  |  |
| Myasthenia (muscle weakness) |  | 1 (11.1) |  |
| Muscle spasm | 2 (9.52) |  |  |
| Paraesthesias | 2 (9.52) |  |  |
| Infection |  |  |  |
| Influenza-like illness | 1 (4.8) | 1 (11.1) | 0.523 |
| Cardiovascular |  |  |  |
| Palpitations |  | 1 (11.1) |  |
| Other |  |  |  |
| Death | 1 (4.8) |  |  |
| Pregnancy | 1 (4.8) |  |  |
| Pruritus | 1 (4.8) |  |  |
| Rash |  | 1 (11.1) |  |
| Pulmonary embolism | 1 (4.8) |  |  |
